# Coarse Grained Molecular Dynamic Simulations for the Study of TNF Receptor Family Members' Transmembrane Organization

**DOI:** 10.3389/fcell.2020.577278

**Published:** 2021-01-21

**Authors:** Mauricio P. Sica, Cristian R. Smulski

**Affiliations:** ^1^Instituto de Energía y Desarrollo Sustentable, Centro Atómico Bariloche, Comisión Nacional de Energía Atómica (CNEA), San Carlos de Bariloche, Argentina; ^2^Medical Physics Department, Centro Atómico Bariloche, Comisión Nacional de Energía Atómica (CNEA), Consejo Nacional de Investigaciones Científicas y Técnicas (CONICET), San Carlos de Bariloche, Argentina

**Keywords:** TNFRSF, tumor necrosis factor receptor superfamily, coarse grained, p75NTR, DR5, Fas (CD95), transmembrane helix assembly

## Abstract

The Tumor Necrosis Factor (TNF) and the TNF receptor (TNFR) superfamilies are composed of 19 ligands and 30 receptors, respectively. The oligomeric properties of ligands, both membrane bound and soluble, has been studied most. However, less is known about the oligomeric properties of TNFRs. Earlier reports identified the extracellular, membrane-distal, cysteine-rich domain as a pre-ligand assembly domain which stabilizes receptor dimers and/or trimers in the absence of ligand. Nevertheless, recent reports based on structural nuclear magnetic resonance (NMR) highlight the intrinsic role of the transmembrane domains to form dimers (p75NTR), trimers (Fas), or dimers of trimers (DR5). Thus, understanding the structural basis of transmembrane oligomerization may shed light on the mechanism for signal transduction and the impact of disease-associated mutations in this region. To this end, here we used an *in silico* coarse grained molecular dynamics approach with Martini force field to study TNFR transmembrane homotypic interactions. We have first validated this approach studying the three TNFR described by NMR (p75NTR, Fas, and DR5). We have simulated membrane patches composed of 36 helices of the same receptor equidistantly distributed in order to get unbiassed information on spontaneous proteins assemblies. Good agreement was found in the specific residues involved in homotypic interactions and we were able to observe dimers, trimers, and higher-order oligomers corresponding to those reported in NMR experiments. We have, applied this approach to study the assembly of disease-related mutations being able to assess their impact on oligomerization stability. In conclusion, our results showed the usefulness of coarse grained simulations with Martini force field to study in an unbiased manner higher order transmembrane oligomerization.

## Introduction

Several reports have shown the importance of pre-ligand assembly of different TNF receptor (TNFR) family members for proper ligand responses (Chan et al., 2000; Siegel et al., 2000; Clancy et al., 2005; Smulski et al., 2013, 2017; Pieper et al., 2014). This ligand-independent association of TNF receptors was initially suggested by the crystal structure of unliganded TNFR1 (Naismith et al., 1995). In that report the authors observed a parallel dimer in which the membrane distal cysteine-rich domain 1 mediated the main interaction interface. This region was not involved in ligand binding and thus seemed to play an exclusive role in pre-ligand assembly. Afterwards, two reports published back to back showed the importance of this region for proper ligand responses for TNFR1 and Fas (Chan et al., 2000; Siegel et al., 2000) and coined the term PLAD for pre-ligand assembly domain. Other reports confirmed these observations and extended it to other TNFR family members (Clancy et al., 2005; Smulski et al., 2013; Pieper et al., 2014). However, whether these associations persist following ligand binding or dissociate to give rise to different ligand-bound structures remains elusive. Moreover, how these oligomeric units (ligand free or ligand bound) impact on the intracellular organization and signal transduction ability, is completely unknown.

The link between extracellular events and intracellular signal transduction is clearly located in the transmembrane region. Thus, getting new insight into the oligomeric properties and the stoichiometry of associations on the transmembrane domains will allow a better understanding of ligand-independent associations, as well as ligand-induced transitions. Nuclear magnetic resonance (NMR) is the method of choice to study the structure and organization of transmembrane segments in a lipid environment. But protein solubility and non-native disulphide oligomerization mediated by free cysteines make this method very cumbersome to apply, especially with short peptides, which have to be mutated as in the cases of p75, Fas, and DR5. Alternatively, atomistic molecular dynamics simulations are suitable to study phenomena at sub-microsecond time-scale involving already formed oligomers of transmembrane segments. However, this approach is computationally infeasible to statistically sample processes at microsecond time-scales with membranes large enough to harbor dozens of individually separated transmembrane helices. Given these limitations, several methods were developed in order to reduce the computational burden of the simulations. Among them, coarse graining the system to a sub-residue level while keeping the relevant chemical properties of the beads, is able to establish a fine balance reaching the necessary sampling and statistical power with reasonable reduction in the detail of the system (Marrink et al., 2007). In addition it is also possible to identify different interfaces responsible for such interactions with sub-residue detail (Bradley and Radhakrishnan, 2013).

In this report we used coarse grained molecular dynamics simulations using the Martini force field to study the transmembrane domain of all available NMR structures of TNFR superfamily (SF) members: p75NTR wt and C257A (TNFRSF16), Fas (TNFRSF6), and DR5 (TNFRSF10B). Each one of these structures showed different association levels such as dimers (Nadezhdin et al., 2016), trimers (Fu et al., 2016), and dimer of trimers (Pan et al., 2019), respectively. Notably, this approach identified similar oligomeric units and similar residues involved in homotypic interactions for most of the simulated structures. This approach allowed to get unbiased information on higher order oligomers which are a key feature for signal transduction in the TNFR superfamily. Moreover, we have tested the impact of different disease related mutations on these associations as well as the differences between the NMR peptide sequences, where free cysteines were replaced by serine, vs. the wild type sequences. This method has proven to be reproducible and robust when compared to NMR data and set the bases for studying other TNFR family members, the impact of pathogenic mutations, different lipid compositions, and/or heteromeric associations.

## Methods

### Coarse Grained Molecular Dynamic Simulations

Coarse-grained (CG) models were built to simulate the interactions of the transmembrane domains of DR5, Fas, and p75 embedded in a lipid bilayer environment solvated with explicit CG water. The CG peptides were constructed using the martinize.py tool (de Jong et al., 2013). The input structures for each helix were obtained from the oligomeric, all-atoms structures determined by NMR for DR5 (PDB: 6nhw), Fas (PDB: 2na7), and p75 (PDB: 2mic). Using pymol, the experimental structures were mutated when necessary to obtain the following input structures: p75 (dimer), Fas (wt), Fas (C178S), Fas (C178R), DR5 (wt), DR5 (A222Y), and DR5 (G217Y) (Table 1). It is worth noticing that Fas (C178S) corresponds to the peptide used in the NMR experiment (Fu et al., 2016).

**Table 1:** Description of the different transmembrane peptides and variants used in the present study, together with the simulation times reached for each peptide.

**TNFR variant**	**Simulation time (μs)**	**Sequence**
p75—NMR (2mic)	–	244-**M**TRGTTDNLIPVYCSILAAVVVGLVAYIAFKRWNS**S**KQNKQ-284
p75 dimer (SS)	6.7	248-TTDNLIPVYCSILAAVVVGLVAYIAFKRWNS**S**-279
p75 (SH)	6.5	248-TTDNLIPVYCSILAAVVVGLVAYIAFKRWNS**S**-279
p75—NMR (2mjo)	–	244-**M**TRGTTDNLIPVY**A**SILAAVVVGLVAYIAFKRWNS**S**KQNKQ-284
p75 (C257A)	8.6	248-TTDNLIPVY**A**SILAAVVVGLVAYIAFKRWNS**S**-279
Fas—NMR (2na7)	–	171-RSNLGWL**S**LLLLPIPLIVWVKRKEVQKT-198
Fas wt	7.3	171-RSNLGWLCLLLLPIPLIVWVKRKE-194
Fas-C178S	9.1	171-RSNLGWL**S**LLLLPIPLIVWVKRKE-194
Fas-C178R	7.3	171-RSNLGWL**R**LLLLPIPLIVWVKRKE-194
DR5—NMR (6nhw)	–	207-**M**P**G**SLSGIIIGVTVAAVVLIVAVFVCKSLLWKKVL-241
DR5 wt	8.6	207-SPCSLSGIIIGVTVAAVVLIVAVFVCKSLLWKKVL-241
DR5-A222Y	8.7	207-SPCSLSGIIIGVTVA**Y**VVLIVAVFVCKSLLWKKVL-241
DR5-G217Y	9.4	207-SPCSLSGIII**Y**VTVAAVVLIVAVFVCKSLLWKKVL-241

*Bold letters indicate mutated residues*.

The starting system consisted of a box of 25 × 25 × 10 nm with 36 individual CG helices evenly spaced in the XY-plane with their axes oriented in the Z axis. The 36 helices were placed in a lipid bilayer on the XY-plane using the INSANE (INSert membrANE) tool, and randomly oriented around Z. The lipids were composed of DOPC and DLPC (7:3) equally distributed on both sides of the membrane. The coarse-grained chain L correlates with 12:0 (lauric) and 14:0 (miristic) saturated fatty acids, whereas chain O correlates with C16:1 (9c) (palmitoleic) and C18:1 (9c) (oleic) unsaturated fatty acids, allowing to build a model of a biological fluid membrane resembling the chain lengths used in NMR experiments. The system was completed with CG water beads and consisted of 36 peptides, 1,700 lipids, 26,000 waters, and 600 ions (150 mM concentration), totalling 48,000 particles. Simulations were carried out with the GROMACS package version 2016.5 (Abraham et al., 2015) using the Martini v2.1 forcefield (Marrink et al., 2007). After the initial steps of minimization and equilibration the systems were simulated with a 20 fs time step at 310 K and 1 bar using the velocity rescaling thermostat of Bussi et al. (2007) and the semi-isotropic Parrinello-Rahman barostat. Every system was simulated for at least 6 μs.

### Contact Maps

For each residue (i) of every helix (H) the number of contacts against all the other residues in the remaining helices, along the simulation time (T) was computed. A contact was defined when the BB atoms of two residues are located at XYZ-distance equal to or less than an arbitrary cut-off, as follows:

$$\begin{array}{l} C_{ij}^{KL}=\{\begin{array}{c} 1,\text{ if}\\ 0,\text{ if} \end{array}\left\|\begin{array}{l} {\text{r}}_{i}^{K}-{\text{r}}_{j}^{H}\\ {\text{r}}_{i}^{K}-{\text{r}}_{j}^{H} \end{array}\right\|\begin{array}{c} \leq d_{\text{cutoff}}\\ >d_{\text{cutoff}} \end{array} \end{array}$$

where i and j are the residue number in the peptide sequence (i = {1,.,j,.,N}), and H and K are the helices analyzed (H = {1,.,K,.,36}). Thus, the number of contacts (NC) for every residue i against each residue j in the remaining (K) helices were computed as:

$$\begin{array}{l} NC_{ij}=\underset{T}{\sum }\sum_{j}^{K\neq H}C_{ij}^{HK} \end{array}$$

We always computed all 36 helices present in the membrane patch against each other. The NMR structures were analyzed considering each model of the PDB file (10 or 15) as a simulation-snapshot. Each individual model was converted to CG model prior to the analysis of contact residues and radial density. We applied two different cut-off distances: 0.5 and 0.8 nm based on the average distance of dimeric or trimeric associations observed in the three NMR structures used as reference in this study. DR5 dimers showed closer interaction interfaces when compared to trimeric assemblies and thus, it was necessary to use two cut-offs distances to fully characterize different assembly modes. Notably, shorter cut-offs distances (0.4 nm) fail to detect any interaction, whereas longer cut-offs distances (1 nm) loose specificity.

### Radial Density

Radial density maps were built to observe the preferential contact side between helices in the XY-plane. First, the centroid (C) of every helix was computed between a defined central backbone (BB) atom (i) and one consecutive BB atom at each side in the sequence (C_i_= (r_i−1_+r_i_+r_i+1_)/3), where r is the *XYZ-*coordinate of the atom (we tested the tool using two BB atoms at each side and observed no significant differences). Second, the unit bisection vector was computed between the central (i) and adjacent BB atoms (i±1), according to the method of Khan to identify the helix orientation (Kahn, 2001). Third, a reference frame was defined with the centroid of the reference helix as origin and its orientation vector as unit X-vector, and the position of the centroids of the remaining 35 helices were computed. This procedure was repeated for all 36 helices present in the membrane patch along the indicated simulation time every 100 ns until the end of the simulated period. The scatter plot of the accumulated XY-centroids positions was transformed to a density map with ggplot implemented in R. This procedure was repeated with every residue along the peptide.

### Symmetry

Symmetry analysis was performed using the Analytical Analyzer of Symmetries software [Ananas (Popov and Grudinin, 2014; Pagès and Grudinin, 2018; Pagès et al., 2018)] using the selected snapshots from the CG simulations.

## Results

### p75NTR (TNFRSF16)

**Figure 1:**
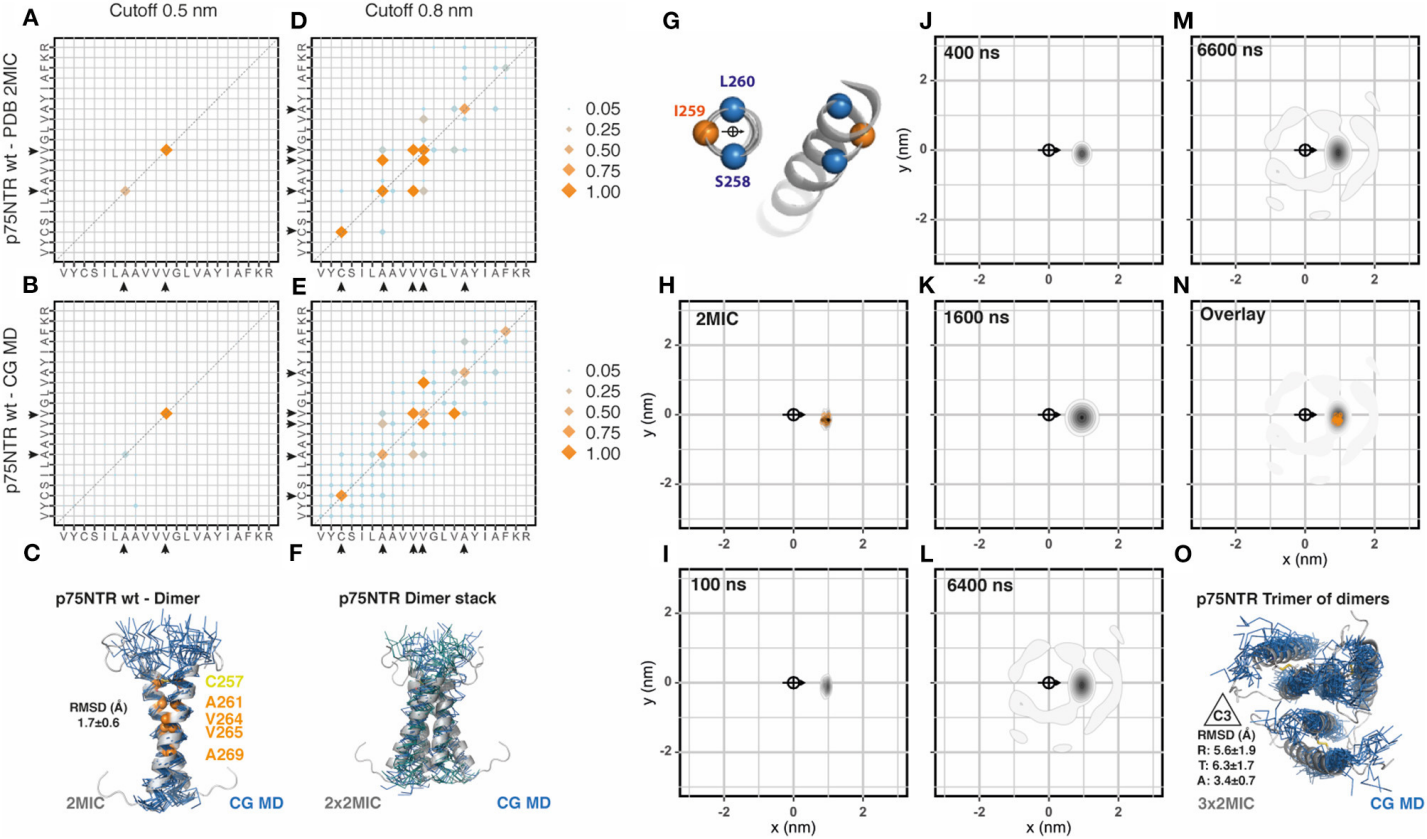
p75NTR assembly. **(A)** Contact matrix of p75NTR wt NMR (PDB:2mic) at 0.5 nm cut-off distance. The amino acid sequence corresponds to residues V254 to R274. Black arrowheads indicate the residues involved in these interactions. Data corresponds to the averaged 10 NMR models. Scales correspond to the number of contacts normalized to the most frequent one [NC_ij_/major(NC_ij_)]. **(B)** Same as **(A)**, for p75NTR coarse grained molecular dynamic (CG-MD) simulation. The analysis was performed on the full system (36x36) between 3 and 6 μs. **(C)** Alignment of the p75NTR NMR average model with a random p75NTR CG-MD dimer, together with the analysis of the averaged root mean square deviation (RMSD) of the alignment. **(D)** Same as **(A)**, but using a cut-off distance of 0.8 nm. **(E)** Same as **(B)**, but using a cut-off distance of 0.8 nm. **(F)** Example of stable interactions between dimers observed along the simulation, aligned to the reference structure 2 mic. **(G)** Residues chosen for the centroid and orientation vector used for radial distribution analysis. **(H)** Radial distribution analysis of p75NTR NMR structure 2mic, analyzed as coarse grained structure. The scale corresponds to the 2D-density function built from the scatter plot of *XY*-coordinates, as described in Methods. **(I–M)** Radial distribution analysis of different snapshots of p75NTR CG-MD at the indicated time points. **(N)** Overlay of the radial distribution of the reference structure (2mic, orange dots) and the CG-MD structures (gray density scale). **(O)** Example of a stable trimer of dimers found at early time points, assembled into the characteristic 3-fold symmetry axes C3, together with the averaged radial (R), tangential (T), and axial (A) RMSD.

Because p75NTR is a covalently linked dimer, we generated a membrane patch and placed 18 evenly distributed disulphide-bonded dimers (36 transmembrane segments) (sequences are shown in Table 1). We extended the coarse-grained (CG) molecular dynamic (MD) simulation to 6.7 μs and compared the output data with the reference NMR structural data (PDB: 2mic) by using the analytic tools described in Methods. We have observed that this and the following simulations converged before 3 μs. In addition, the area-per-lipid and membrane thickness also converged to the standard values of 0.73 nm^2^ and 3.6 nm, respectively. We first evaluated the residues involved in helix-to-helix interactions between the 36 helices integrating all data points from the third μs of simulation until the end of the simulated period. To this end, we generated a contact matrix of the residues closer than 0.5 nm for the NMR and the CG-MD simulated data (Figures 1A,B, respectively, and Supplementary Figure 1) which resulted in identical contact residues. These residues were located on the dimeric interface of the NMR structure at the crossing point (AxxxV). To further compare the similarity between the dimeric structure obtained by the two approaches, we aligned the NMR dimeric structures with the CG-MD structures backmapped to all atom structures as described in Wassenaar et al. (2014) and observed an average root mean square deviation (RMSD) value of 1.7 ± 0.6 Å (Figure 1C). Similar results were observed when analyzing the 0.8 nm cut-off but with a few additional contact points toward the C- and N-terminal regions for both NMR and CG simulations (Figures 1D,E). The residues observed in the 0.8 nm cut-off radius included the two residues observed in the 0.5 nm cut-off, indicating that both contact matrices are showing the same interaction interface. In addition to the main dimeric association, we observed several dimers stacks in a very conserved parallel arrangement (Figure 1F). In order to better characterize the dynamics of the spatial distribution of p75NTR dimers, we then analyzed the radial distribution around each one of the 36 helices present in the membrane patch against each other at different time points along the simulation period. The orientation was determined by the residues S258, I259, and L260 which were also used to determine the center of reference (Figure 1G). We performed this analysis on the 10 coarse-grained NMR (CG-NMR) models available in the 2mic PDB structure (Figure 1H) and, as expected, we observed only one position corresponding to the covalent dimer. When we applied this analysis to the CG-MD simulation, we observed a main spot corresponding to the covalent dimer at early time points (Figures 1I–K). Notably, higher order associations between dimers were observed at later time points (Figures 1L,M). The overlap of the radial distribution of the CG-NMR structure with the CG-MD simulation showed that the covalently linked dimers are exactly on the same relative position in the radial map (Figure 1N). Amongst the higher order associations formed during the CD-MD, we observed a stable trimer of dimers with the characteristic 3-fold symmetry axes (C3) (Figure 1O). Using the C3 relative orientation as cutoff criteria we quantified four different disulphide linked dimers, with a mean association time of 325 ns, present at different time points. This observation is compatible with the trimeric organization observed in Fas and DR5, which is notably conserved across the structure of TNF superfamily ligands and signal adaptor molecules TRAFs. The evolution of the CG-MD simulation is shown in Supplementary Movie 1.

Experimental data showed that, under reducing conditions, p75NTR wt is in a monomer-dimer equilibrium with the C257 residue located on the dimeric interface (Nadezhdin et al., 2016). We therefore also simulated a reduced version of p75NTR wt and compared the results to another available NMR structure (2mjo) corresponding to the functionally inactive p75NTR C257A mutant, which shows a left handed dimer through the AxxxG motif located on the opposite face of the α-helix (Supplementary Figure 1). Both simulations showed a rather diffuse contact matrix when using a cut-off of 0.8 nm, which may suggest a diversity of configurations. These matrices do, however, include the contacts observed in the NMR structures. Notably, both CG-MD simulations showed many similarities between them regarding the radial distribution, which is in agreement with the lack of disulphide bonds between helices (Supplementary Figure 1B, bottom panels). Moreover, there were two visible spots (among others) in each simulation (reduced p75NTR and C257A) that overlapped partially with the corresponding dimers observed in the two NMR reference structures (Supplementary Figure 1B, bottom panels, orange and blue dots). Nevertheless, only the spots located close to the disulphide-linked-like region (Supplementary Figure 1B, bottom panels, orange dots) showed a main relative orientation of ~180° for both non-dimeric structures (reduced p75NTR and C257A). Although these results do not match the NMR reference structure (2mjo), they could arise from differences between lipid phases since NMR experiments were made in detergent micelles and our simulation in phospholipid bilayers.

### Fas (TNFRSF6)

**Figure 2:**
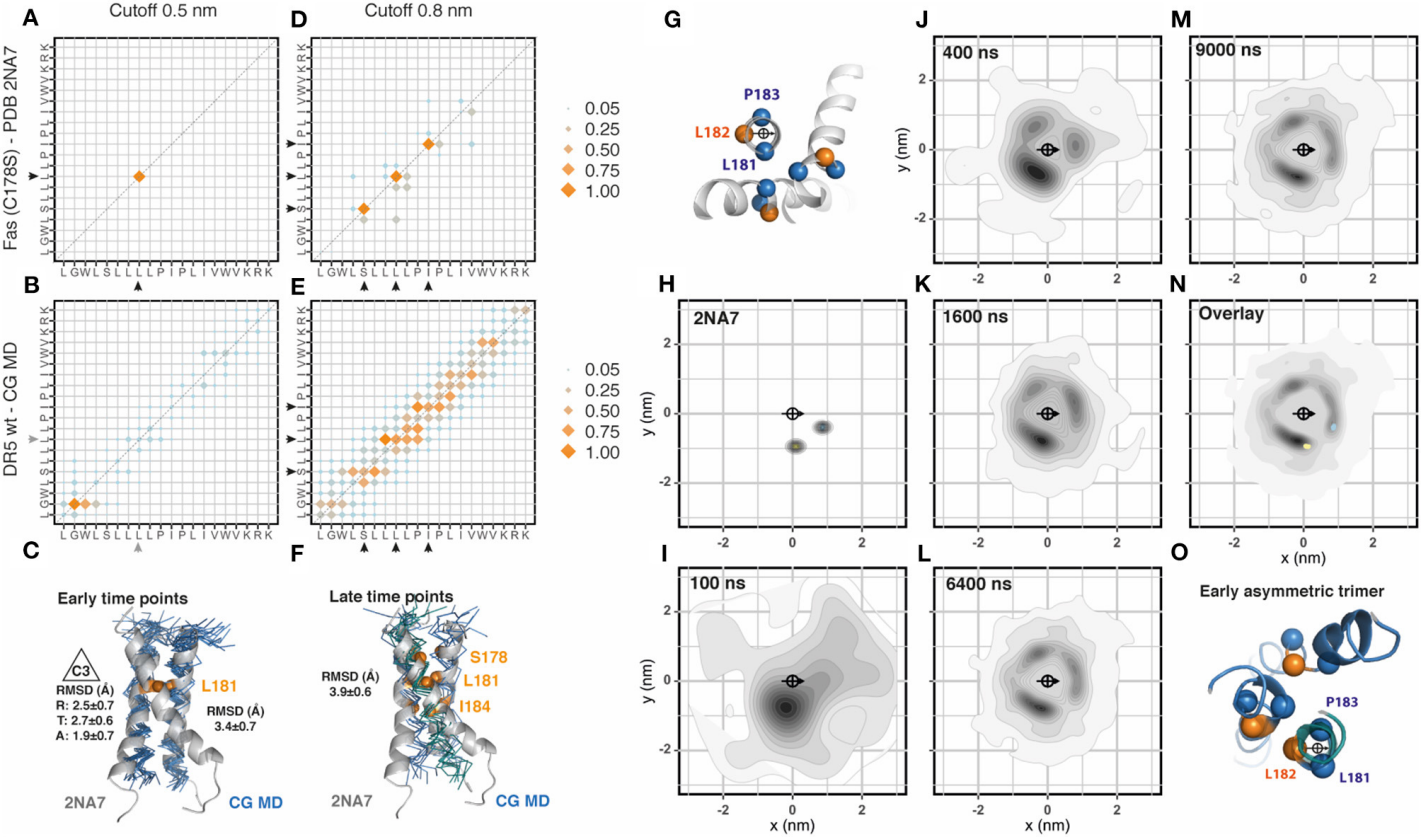
Fas assembly. **(A)** Contact matrix of Fas C178S NMR (PDB:2na7) at 0.5 nm cut-off distance. The amino acid sequence corresponds to residues L174 to K193. Black arrowheads indicate the residues involved in these interactions. Data corresponds to the averaged 15 NMR models. Scales correspond to the number of contacts normalized to the most frequent one. **(B)** Same as **(A)**, for Fas C178S CG-MD simulations. Gray arrowheads indicate non-conserved interactions observed only in the simulation. The analysis was performed on the full system (36 × 36) between 3 and 6 μs. **(C)** Alignment of the Fas NMR average model with a Fas CG-MD dimer arranged in a 3-fold symmetry axes (C3). This structure is formed at early simulation times (0.5–1 μs). The averaged root mean square deviation (RMSD) of the alignment is indicated on the right and the averaged radial (R), tangential (T), and axial (A) RMSD is indicated on the left. **(D)** Same as **(A)**, but using a cut-off distance of 0.8 nm. **(E)** Same as **(B)**, but using a cut-off distance of 0.8 nm. **(F)**. Alignment of the Fas NMR average model with a Fas CG-MD trimer, together with the analysis of the averaged root mean square deviation (RMSD) of the alignment. This structure is formed at late time points (5–6 μs). **(G)** Residues chosen for the centroid and orientation vector used for radial distribution analysis. **(H)** Radial distribution analysis of the coarse grained Fas C178S NMR structure 2na7. The scale corresponds to the 2D-density function from the scatter plot, as described in Methods. **(I–M)** Radial distribution analysis of different snapshots of Fas C178S CG-MD at the indicated time points. **(N)** Overlay of the radial distribution of the reference structure (2na7, blue/yellow dots) and the CG-MD structures (gray density scale). **(O)** Example of an early trimer, where the third helix assembles in an asymmetric manner, generating the visible spot on top of the central helix.

Different from p75NTR, Fas NMR structure showed a trimeric assembly. We followed the procedure previously described and inserted 36 Fas transmembrane segments evenly distributed in the membrane patch. The Fas sequence used for the simulation corresponded to the Fas variant C178S used for the NMR structure (PDB: 2na7) as shown in Table 1. The analysis of the residues involved in helix-to-helix contacts using a cut-off distance of 0.5 nm showed very few contact residues, which is explained by the relative distance between the helices forming the trimeric assembly (Figures 2A,B, Supplementary Figure 2). Initially, mainly dimeric associations were observed. These dimers were placed in two main orientations compatible with a two-fold symmetry axis (~25%) and with a 3-fold symmetry axis (C3, ~19%) allowing the late inclusion of the third helix of the trimer. When aligned to the NMR structure, these dimers showed an average RMSD value of 3.4 ± 0.7 Å (Figure 2C). The analysis of the 0.8 nm cut-off distance showed very well-conserved residues. However, these residues seem to be rather flexible in the CG-MD, most probably due to the late formation of the complete trimeric unit or to the presence of alternative assembly modes using the same interfaces (Figures 2D,E). At later time points, it is possible to identify two trimeric assemblies which resemble the NMR structure (Figure 2F). Then, we analyzed the radial distribution around each one of the 36 helices present in the simulation against each other. The orientation was determined by the residues L181, L182, and P183 which were also used to determine the center of reference (Figure 2G). We performed this analysis for the CG-NMR structures computing the 15 different models available in the NMR structure file (PDB: 2na7) (Figure 2H). As expected, we observed two main positions corresponding to the trimeric assembly. When we applied this analysis to the CG-MD simulation we observed a main spot corresponding to the trimer-compatible dimer and two other, less strong signals at early time points (Figures 2I–K). At later time points, the second trimer-compatible spot starts to get defined (Figures 2L,M). The overlap of the radial distribution of the CG-NMR structure with the CG-MD simulation showed that the NMR trimer position corresponds to two out of the three spots observed in the CG-MD simulation. The third spot, located in the upper left side of the central helix corresponded to the asymmetric helix of the trimer when it is located at the center of the quadrant (Figures 2N,O). Using a clustering approach to isolate the main NMR-like cluster, we found an average of 26.2 ± 1.3 C2 dimers that were formed between 28 different transmembrane helices. The accumulated association time was 32.5 ± 1.3 μs and the most stable associations extended for over 5.4 μs. The combined analysis of trimeric assemblies on the two NMR-like clusters showed an average of 33.6 ± 1.2 C3 dimers that were formed between 34 different transmembrane helices. However, the accumulated association time was lower than C2 dimers (16.7 ± 1.1 μs), being the most stable association 3.8 μs. All together, these results suggested that Fas trimeric assembly during the CG-MD simulation might occur in at least three steps characterized by the initial assembly of a trimer-compatible dimer, the association of an asymmetric third helix (which produces the third spot on the top-left side of the central helix) and the re-placement of this third helix (Figure 2O). However, we could not find any inverse correlation between the amount of dimers and trimers along the simulated period. The evolution of the CG-MD simulation is shown in Supplementary Movie 1.

One drawback of NMR methodology is the presence of cysteine residues in the peptide sequence. The side chain of free cysteines is highly reactive and affects the solubility of the peptide so it is frequently replaced by serine. This was the case of Fas NMR reference structure (PDB: 2na7, C178S). We therefore generated and simulated the wt sequence of Fas as well as a pathogenic mutation C178R located at the same residue mutated in the NMR structure, which is associated with cutaneous squamous cell carcinoma (Lee et al., 2000). Similar to Fas C178S, Fas wt showed early time point stable dimers. However, these dimers were formed in a different position, which correspond to the asymmetric third helix observed in Fas C178S CG-MD (Supplementary Figure 2). The main NMR-like spot was severely reduced in these two structures (Fas wt and C178R) but in a different manner: while Fas wt showed an average of 27.6 ± 1.2 C2 pairs with an accumulated association time of 13.9 ± 0.7 μs, Fas C178R showed an average of 16.2 ± 0.9 C2 pairs with an accumulated association time of 7.0 ± 0.3 μs. These observations suggest that the main NMR-like spot is severely affected by these two mutations which are facing in that direction. Comparing Fas wt with Fas C178R we observed similar distribution patterns although the main spots of Fas C178R were rotated anti-clockwise when compared to Fas wt. Interestingly, we observed conserved numbers of C3 compatible dimers in Fas wt (35 ± 0.7) with an accumulated association time of 18.6 ± 1.1 μs, which is in clear contrast to Fas C178R that showed an average of 24.6 ± 1.2 C3 pairs with an accumulated association time of 9.1 ± 1.2 μs. We didn't observe any stable trimer formation in these two structures. Importantly, Fas wt contact matrix showed a rather organized assembly with three main contacts that differ from Fas C178R, indicating that the mutation alters the interaction interfaces thereby changing the geometry of the assembly (Supplementary Figure 2).

### DR5 (TNFRSF10B)

**Figure 3:**
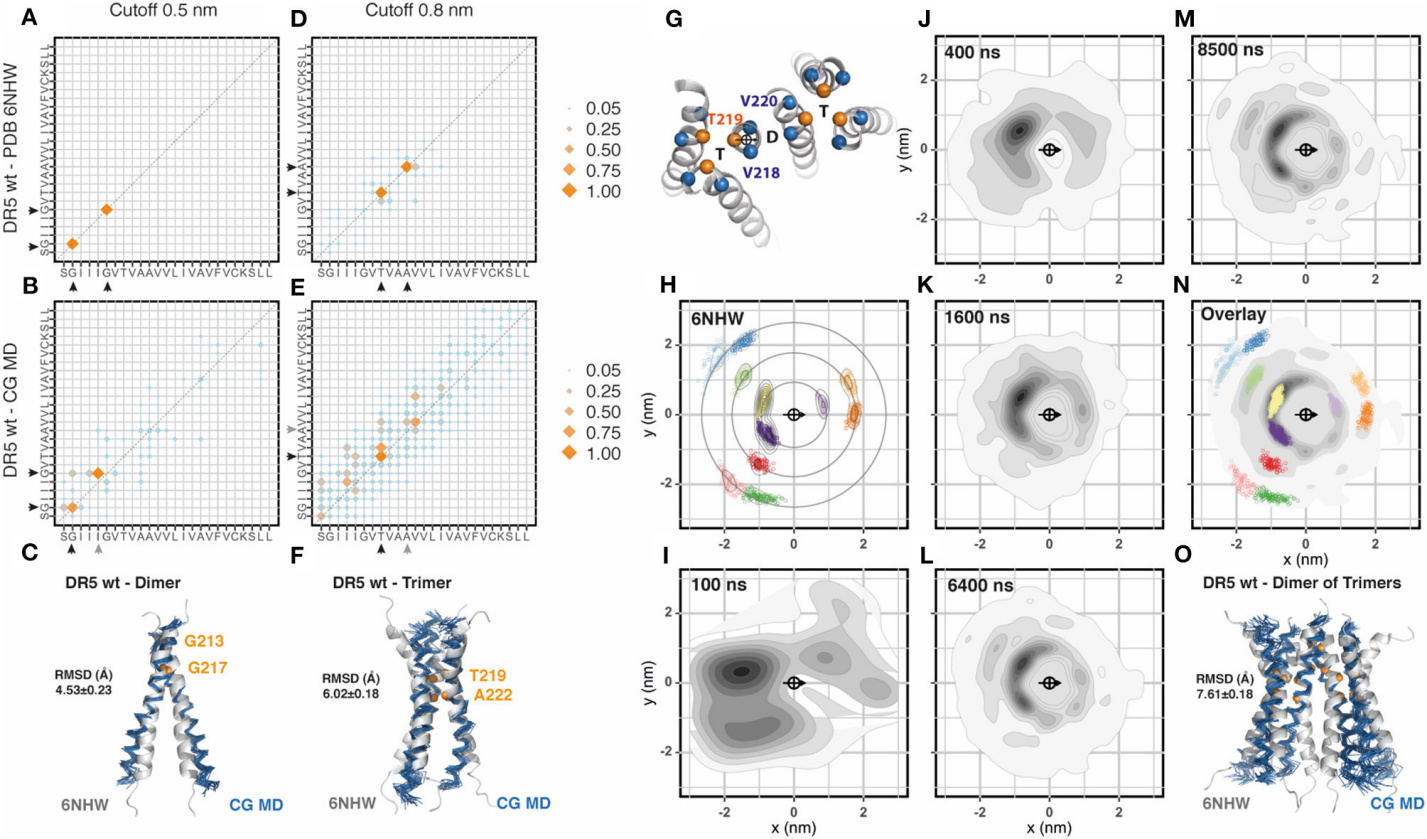
DR5 assembly **(A)**. Contact matrix of DR5 NMR (PDB:6nhw) at 0.5 nm cut-off distance. The amino acid sequence corresponds to residues S212 to L236. Black arrowheads indicate the residues involved in these interactions (GxxxG motif). Data corresponds to the averaged 15 NMR models. Scale corresponds to the number of contacts normalized to the most frequent one. **(B)** Same as **(A)**, for DR5 coarse grained molecular dynamic (CG-MD) simulations. Black arrowheads indicate conserved interactions whereas gray arrowheads indicate non-conserved interactions. The analysis was performed on the full system (36 × 36) between 3 and 8 μs. **(C)** Alignment of DR5 NMR average dimer with a DR5 CG-MD dimer, together with the analysis of the averaged root mean square deviation (RMSD) of the alignment. **(D)** Same as **(A)**, but using a cut-off distance of 0.8 nm. **(E)** Same as **(B)**, but using a cut-off distance of 0.8 nm. **(F)** Alignment of DR5 NMR average model with a DR5 CG-MD trimer, together with the analysis of the averaged root mean square deviation (RMSD) of the alignment. **(G)** Residues chosen for the centroid and orientation vector used for radial distribution analysis. **(H)** Radial distribution analysis of coarse grained DR5 NMR structure 6nhw. Note that it is possible to identify dimers, trimers, and higher order assemblies in consecutive orbits. Scale corresponds to the 2D-density function from the scatter plot, as described in Methods. **(I–M)** Radial distribution analysis of different snapshots of DR5 CG-MD at the indicated time points. **(N)** Overlay of the radial distribution of the reference structure (6nhw, color dots) and the CG-MD structures (gray density scale). **(O)** Alignment of DR5 NMR average dimer of trimers with a DR5 CG-MD assembly, together with the analysis of the averaged root mean square deviation (RMSD) of the alignment.

The most recently published transmembrane NMR structure of a TNFRSF member corresponds to DR5 (Pan et al., 2019). In this structure (PDB: 6nhw) it is possible to observe a dimer of trimers, which is the most complex assembly described so far for the transmembrane region of a TNFRSF member. We followed the same procedure previously described and inserted 36 DR5 transmembrane segments evenly distributed in a membrane patch. The sequence corresponded to DR5 wt, which differs from the mutated version used for NMR (C209G) as shown in Table 1. The analysis of helix-to-helix residue contacts using a cut-off distance of 0.5 nm showed the same dimeric interface observed in NMR experiments (Figures 3A,B, Supplementary Figure 3). There were some minor differences in the pairing of the GxxxG motif known for mediating transmembrane helix dimerization but not trimerization (MacKenzie et al., 1997; Trenker et al., 2015). The reasons for these deviations may be multiple: i.e., a slight change in tilt can prevent the contact between two glycine and slide this contact one position, especially if the glycine is flanked by two bulky residues as Ile and Leu. Next, we aligned the NMR dimer with the CG-MD structure and observed stable dimers that match the reference structure with an averaged RMSD of 4.53 ± 0.23 Å (Figure 3C). The analysis of the 0.8 nm cut-off distance showed very well-conserved residues with some minor differences toward the C-terminal region of the interaction interface (Figures 3D,E). Note that the residues observed at 0.5 and 0.8 nm cut-off were different and corresponded to the dimeric and trimeric assembly, respectively. The alignment of the NMR trimer to the CG-MD trimers showed an averaged RMSD of 6.02 ± 0.18 Å (Figure 3F). Then, we analyzed the radial distribution around each one of the 36 helices present in the simulation against each other. The orientation was determined by the residues V218, T219, and V220 which were also used to determine the center of reference (Figure 3G). We performed this analysis for the CG-NMR structure computing the 15 different models available in the NMR structure file (Figure 3H). As expected, we observed the full landscape of associations, namely dimers, trimers and dimers of trimers in consecutive orbits around the central helix. When we applied this analysis to the CG-MD simulation at early time points, we observed two main spots corresponding to one of the trimeric units and one clearly distinct spot corresponding to the dimer (Figures 3I–K). As the simulation proceeds, the second trimeric spot starts to get defined together with higher order oligomers present in consecutive orbits around the central helix (Figures 3L,M). The overlap of the radial distribution of the CG-NMR structure with the CG-MD simulation showed a striking similar distribution, even in regions far away from the central helix (Figure 3N). These results indicate that CG-MD simulation of DR5 transmembrane domain can identify the characteristic dimer of trimers observed in NMR studies (Figure 3O). However, there were a few unidentified spots around the central helix that could not be assigned to dimers or trimers. Using a clustering approach to isolate the main spots on the first orbit (<1.5 nm) it was possible to analyse the distance and relative orientations of each cluster (Supplementary Figures 4A,B). We used two reference residues to study the dimeric (G217) and the trimeric (T219) assembly matching the contact matrix (Supplementary Figures 4C,D). Each cluster was isolated and analyzed in a comparative manner against the expected NMR distribution and against each other cluster for distance, radial location (alpha), and relative orientation (beta) (Supplementary Figures 4C,D). This analysis showed that the dimeric cluster although being less populated than others can be clearly identified by its proximity to the central helix and by its relative (beta) orientation, close to 180° (Supplementary Figure 4C). The trimeric assembly was clearly more populated but also showed distinct features that differentiate them from the neighbor spots. They showed a closer proximity to the central helix and a relative orientations closely matching the expected C3 relative orientation of 120 and 240° (beta) (Supplementary Figure 4D). The remaining spots showed complex mixed compositions in terms of relative orientations. We generated a Markov chain model with the trajectories along the radial clusters which showed that the unidentified spots travel toward the neighbor main spots with relatively high probability. Also, the probability of remaining in the same cluster is higher for the dimeric and trimeric spots (Supplementary Figure 4E). Using the clustering approach and the relative orientation criteria we observed an average of 17.8 ± 1 C2 dimers that were formed between 28 different transmembrane helices. These dimers showed an accumulated association time of 18.1 ± 0.9 μs (Supplementary Figure 4C). Additionally, there was an average of 11.3 ± 1 C3 trimers that were formed between 27 different transmembrane helices. These trimers showed an accumulated association time of 25.3 ± 1.8 μs (Supplementary Figure 4D). The evolution of the CG-MD simulation is shown in the Supplementary Movie 1.

Based on their NMR structure, Pan and colleagues (Pan et al., 2019) introduced two different mutations into the DR5 transmembrane sequence to disrupt dimeric (G217Y) or trimeric (A222Y) interactions. Therefore, we performed a CG-MD simulation for each one of these DR5 mutants and compared them to the wt sequence. Mutation G217Y, aimed at disrupting dimers, showed conserved trimeric spots but reduced dimeric spots in the radial distribution plots. The contact matrix confirmed the impact of the G217Y mutation on the dimerization face but also showed some differences in the trimerization face. As expected, the analysis of the dimerization rate showed reduced number of dimers with reduced association times. However, despite showing conserved NMR-like trimeric spots, the trimerization rate was also affected due to a wider distribution of the relative orientations (beta angles) of the helices when compared to wt DR5 simulation (Supplementary Figure 4D). Mutation A222Y, aimed at disrupting trimers, showed a clearly conserved dimeric spot while the two trimeric spots were fused into one strong signal in between the two wt positions (Supplementary Figure 3). The clustering analysis confirm this observation, showing a conserved dimeric assembly and a strongly impaired trimeric assembly (Supplementary Figure 4). Our results indicate that CG-MD simulation of DR5 transmembrane region recapitulates the main features described for the wt sequence like dimerization, trimerization, and the complex dimer of trimer assembly. Additionally, our results showed a broader impact of the specific mutations that were described to affect exclusively dimeric or trimeric associations.

## Discussion

Since the report of the first structure of the extracellular domain of the unliganded tumor necrosis factor receptor (Naismith et al., 1995), the TNF-related scientific community is interested in understanding the role of ligand independent receptor assembly in signal transduction. Naismith and colleagues showed that the soluble extracellular domain of TNFRSF1A was able to form homodimers in the absence of ligand and opened the discussion of whether these dimers restrain the receptor in an inactive ligand-free state or if they persist following ligand binding to extend an activating network (Naismith et al., 1996). Because TNF family ligands are trimeric molecules and signal adaptor molecules of the TNFR associated factors (TRAF) group are also trimeric proteins it seems possible that ligand independent dimers represented a “silent” receptor form. Several reports confirmed the occurrence of extracellular, ligand-independent associations, and its importance for proper ligand binding and signal transduction (Chan et al., 2000; Siegel et al., 2000; Clancy et al., 2005; Smulski et al., 2013; Pieper et al., 2014). However, such a model cannot be extended to small TNFR superfamily (TNFRSF) members which do not possess a pre-ligand assembly domain, and also it does not explain the impact of pathogenic mutations located in the transmembrane region of several TNFRSF members. Recent reports showed the active role of the transmembrane domains to stabilize homotypic interactions in different TNFRSF members, participating actively in signal transduction (Fu et al., 2016; Nadezhdin et al., 2016; Pan et al., 2019). These studies used the NMR technique to obtain structural information on the transmembrane domain organization. So far, 3 out of 30 TNFRSF members transmembrane regions have been studied by NMR and each one of them showed different association patterns: p75 assembles as a covalent dimer, Fas assembles as a trimer, and DR5 assembles as a dimer of trimers. Unfortunately, such differences between available structural data make it impossible to generalize any kind of conserved molecular determinants, pattern, or interaction motif. Moreover, NMR studies are complex and expensive and it is thus unlikely that sufficient data will be obtained on the remaining TNFRSF to conclude on the physiological function of their transmembrane associations or the impact of disease-associated mutations in the transmembrane region.

There are a few available methods to perform structural modeling of TM α-helical with the limitation that most of them are restricted to the simulation of dimers: PREDDIMER (Polyansky et al., 2014), CATM (Mueller et al., 2014), EFDOCK-TM (Wang and Barth, 2015), or TMDOCK (Lomize and Pogozheva, 2017). However, TNFRSF members seem to associate as higher order oligomers such as trimers, or dimers of trimers. To be able to explore such complex level assemblies, we used CG-MD simulations which allowed us to explore oligomerization as a dynamic process occurring at the microsecond time scale, which would be impossible with atomistic simulations (Bradley and Radhakrishnan, 2013). Given the diversity of structures observed in these three NMR models, we could assess the potential and shortcomings of CG-MD simulations to study different transmembrane association modes in different TNFR superfamily members.

There are a few reports on the use of coarse-grained molecular dynamic simulations to study dimeric, trimeric or tetrameric assemblies. However, most of them just place in their membranes the exact number of helices that they want to study (Hall et al., 2014; Wassenaar et al., 2015; Han et al., 2016) (biased approach), or several copies with the aim of characterizing just one kind of association (i.e., dimers) (Periole et al., 2012). In order to allow the unbiassed formation of complex oligomeric arrays and increase the statistical sampling of our results, we introduced 36 evenly distributed and randomly oriented helices and let the system evolve for a time frame of at least 6 μs. The membranes were built with phospholipids of fatty acid length and head groups similar to the ones used in NMR experiments. To consolidate the unbiased approach, we analyzed all 36 helices against each other for close contact residues and relative positions of neighbor helices and compared the results to the corresponding structural data available.

Our data using p75NTR sequence (disulphide linked dimers) showed a striking similarity when compared to the PDB 2mic, both at the level of residues involved in helix to helix interactions and at the observed radial distribution. In addition, we could observe some higher order oligomers, dimers stacks and an intriguing trimer of dimers with a stable 3-fold symmetry axes (C3) along the simulation. These higher order complexes were less prominent than the covalently linked dimers and therefore, their detection was not evident in the radial distribution analysis. Whether these associations are of functional relevance need to be assessed under specific experimental conditions. Notably, the analysis of p75NTR C257A variant, despite being very similar to the reduced p75NTR wt form, did not match the NMR reference model (2mjo). However, NMR experiments with p75NTR used micelles of dodecyl phosphocholine detergent which might not mimic properly the lateral diffusion of plasmatic membranes as reported by a study on integrins that form dimers in detergents but oligomers in liposomes (Yu et al., 2015).

The analysis of Fas showed a few differences when compared to NMR data. Initially, mainly dimeric associations were observed placed in one of the expected NMR trimeric spots. These dimers were placed in a range of different orientations, being the main ones a two-fold (~180°) and a 3-fold (~120°) symmetry axis. Toward the latest time points of the simulation it was possible to observe slowly forming NMR-like trimers. These, behavior could arise from our simulation conditions. Longer simulation times or higher helix concentrations may be necessary to properly sample this system and approach reasonably to the equilibrium. It is noteworthy that Fu et al., proposed that the inactive receptor form corresponded to a dimer whereas the active form corresponded to the trimer and, thus, the NMR trimer may reflect the active receptor structure (Fu et al., 2016). Unfortunately, they did not provide any structural information on the dimeric assembly. Still, it is tempting to speculate that the inactive dimer may correspond to the incomplete trimer, which is formed in a C2 symmetry, ready to be reoriented in a C3 symetry and allow the inclusion of a third helix following ligand binding.

Because Fas NMR experiments were performed with Fas C178S, we simulated the wt sequence and a pathogenic mutation located in the same residue C178R (Lee et al., 2000). Intriguingly, Fas wt did not fully reproduce Fas C178S behavior but showed an alternative assembly mode forming mainly dimers. This seemingly discrepancy can be due to the impact of the mutation itself, to artifacts during the CG-MD simulations or could be a consequence of the lipid environment. Indeed, NMR studies were carried out in bicelles composed of homo-diacyl glycerophosphocholines with myristic fatty acid and hexanoic acid that might not reproduce the properties of a biological bilayer (Nadezhdin et al., 2016). Despite these differences, residue 178 is located toward the trimeric contact face and, although the Cys-to-Ser replacement implies only one atom, both residues have remarkable differences regarding their hydrophobicity, which may impact on wt-like associations.

The analysis of DR5 showed remarkable similarities when compared to the NMR structural data. This was the case for the contact residues involved in dimeric and trimeric interactions and also for the radial distribution. We could identify dimers, trimers and a dimer of trimers and the radial distribution showed conserved positions across several orbits beyond the central helix. However, the analysis of the mutation G271Y and A222Y showed not only altered dimeric and trimeric assembly, respectively, as it was described before (Pan et al., 2019), but also changes in the relative orientations of the remaining associations that were supposed to be unaffected. Despite the sequence of DR5 used for NMR studies was C209G, we used the wt sequence for CG-MD without observing major differences, most probably due to the fact that this residue was located in the extracellular interface and did not participate in any helix-helix interaction.

There are several types of *post-hoc* analysis that can be applied to the data depending on specific biological questions. In this study we systematically compared our observations to the corresponding NMR structures to validate the use of coarse-grained molecular dynamic simulations to study TNFR superfamily members. Among the several possible analyses, data can be filtered using geometrical criteria for dimers, trimers or more; or analyse the relative position of the spots around the central helix (alpha angle) vs. the relative orientation of the helices in each spot toward the central helix (beta angle); or several other analysis that may arise from specific questions that want to be explored in the system. In this manuscript we used a combination of these analysis as illustrated in Supplementary Figure 4.

Some reports have pointed out that Martini force field overestimates intermolecular interactions of peptides and proteins in membranes (Javanainen et al., 2017) and in solution (Stark et al., 2013). Thus, the system gets trapped in interactions that hardly dissociate and this reduces the power of sampling. However, in this study, Martini force field reproduced the vast majority of association modes and oligomeric levels observed in all NMR reference structures. Moreover, once equilibrated, the helices are distributed in separated clusters and various association-dissociation events occur. Still, non-covalent dimers were more difficult to detect than trimers or higher order oligomers because of the presence of native and non-native interactions, which could indicate that CG-MD simulation may be not optimized for low affinity associations or that these interactions require longer exploration times. We expect that this method gains robustness with the new releases of the Martini force field. In addition, analyzing more NMR solved single span transmembrane proteins, will lead to a better understanding of the weaknesses and strengths of the method.

In summary, we have validated the use of CG with Martini force field to study the oligomerization of TNFRSF members by comparing our results to the available NMR structures, and we have extended this application to assess possible structural changes related to disease-associated mutations. Our study paves the way to analyse the transmembrane organization of different TNFRSF members and other single span transmembrane receptors in a dynamic mode along extended simulation times. The flexibility of the system allows to simulate and study the impact of lipid composition (high vs. low cholesterol and glycosphingolipids or asymmetric lipid compositions), post-translational modifications (such as palmitoylation) as well as heterotypic interaction with other integral membrane proteins.

## Data Availability Statement

The original contributions generated for the study are included in the article/Supplementary Material, further inquiries can be directed to the corresponding author/s.

## Author Contributions

All authors listed have made a substantial, direct and intellectual contribution to the work, and approved it for publication.

## Conflict of Interest

The authors declare that the research was conducted in the absence of any commercial or financial relationships that could be construed as a potential conflict of interest.
