# Supplementary material for: Coarse Grained Molecular Dynamic Simulations for the Study of TNF Receptor Family Members' Transmembrane Organization
Source: Front Cell Dev Biol. 2021 Jan 21;8:577278. doi: 10.3389/fcell.2020.577278 (PMC7859260; doi:10.3389/fcell.2020.577278)
Supplement: Supplementary file 1 [file Data_Sheet_1.pdf]

# Coarse grained molecular dynamic simulations for the study of TNF receptor family members' transmembrane organization.

Mauricio P. Sica [1,2] and Cristian R. Smulski [2]
[1] Instituto de Energía y Desarrollo Sustentable, Centro Atómico Bariloche, Comisión Nacional de Energía Atómica (CNEA), Av. E. Bustillo 9500, R8402AGP San Carlos de Bariloche, Río Negro, Argentina.
[2] Medical Physics Department, Centro Atómico Bariloche, Comisión Nacional de Energía Atómica (CNEA), Consejo Nacional de Investigaciones Científicas y Técnicas (CONICET), Av. E. Bustillo 9500, R8402AGP San Carlos de Bariloche, Río Negro, Argentina.

## SUPPLEMENTARY FIGURES AND FIGURE LEGENDS

**Supplementary movie 1 |** Time course animation of coarse grained molecular dynamics simulations.

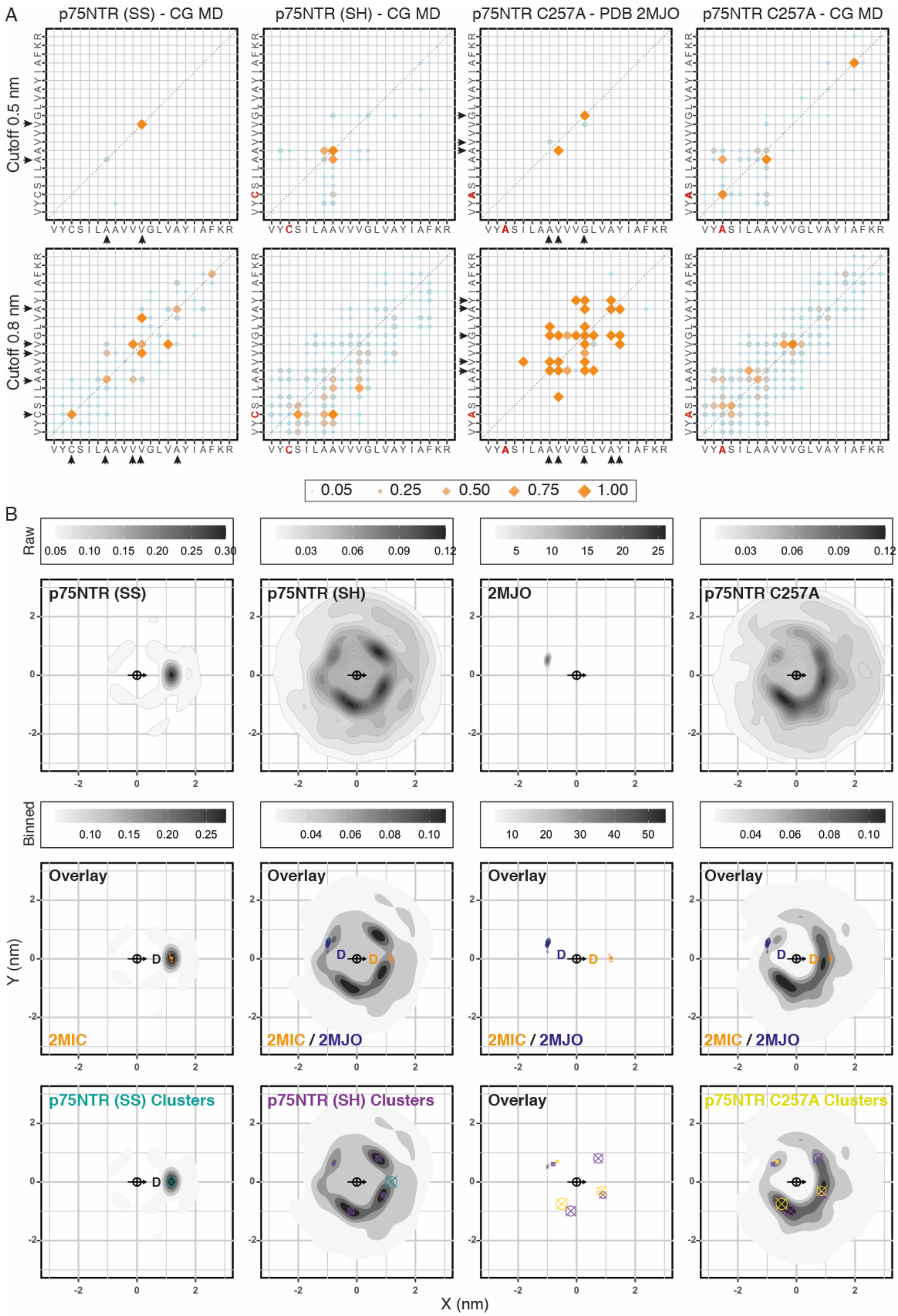
A
p75NTR (SS) - CG MD
p75NTR (SH) - CG MD
p75NTR C257A - PDB 2MJO
p75NTR C257A - CG MD
Cutoff 0.5 nm
Cutoff 0.8 nm
VYCSILAAVVVGLVAYIAFKR
VYASILAAVVVGLVAYIAFKR
0.05
0.25
0.50
0.75
1.00
B
Raw
Binned
p75NTR (SS)
p75NTR (SH)
2MJO
p75NTR C257A
Overlay
2MIC
2MIC / 2MJO
p75NTR (SS) Clusters
p75NTR (SH) Clusters
p75NTR C257A Clusters
D
Y (nm)
X (nm)

**Supplementary Figure 1 |** p75NTR assembly. **(A)** Comparison between the interaction matrix of the coarse grained molecular dynamic (CG-MD) simulations of p75NTR disulphide linked (SS), p75NTR reduced cysteine (SH), p75NTR C257A NMR structure 2mjo and the corresponding CG-MD p75NTR C257A variant at 0.5 and 0.8 nm cut-off distance. The amino acid sequence corresponds to residues V254 to R274. Black arrowheads indicate conserved interactions whereas grey arrowheads indicate non-conserved interactions. Analysis was performed over the 10 NMR models and between 3 to 6 µs in the CG-MD simulations. Scale corresponds to normalized number of contacts. **(B)** Comparison of the radial distribution of coarse grained p75NTR disulphide linked (SS), p75NTR reduced cysteine (SH), p75NTR C257A NMR structure 2mjo and the corresponding CG-MD p75NTR C257A variant. Top panels showed raw data. Middle panels show binned data, to allow better visualization of the main distribution spots. The dimeric assembly (D) of p75NTR NMR (2mic) is represented as orange dots whereas dimeric assembly (D) of p75NTR C257A NMR (2mjo) is represented as blue dots. Lower panels show the same binned data in which it was overlapped the main dots observed in the CG-MD models p75NTR (SS) (green), p75NTR (SH) (violet) and p75NTR C257A (yellow). In all cases the size of the centred circles correspond to the amount of data points in each cluster. Scales correspond to the 2D-density function from the scatter plot *XY*-coordinates.

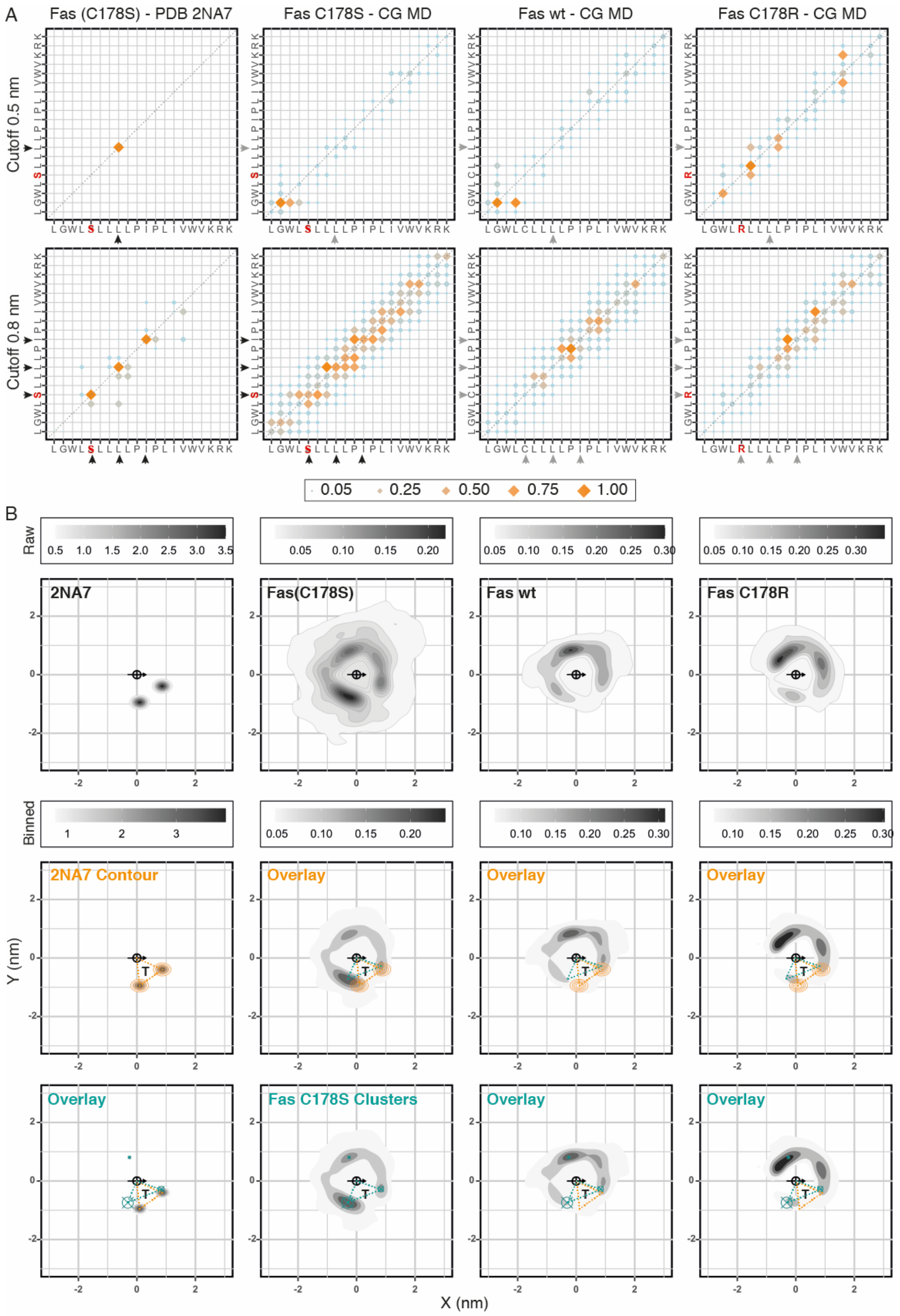
A
Fas (C178S) - PDB 2NA7
Fas C178S - CG MD
Fas wt - CG MD
Fas C178R - CG MD
Cutoff 0.5 nm
Cutoff 0.8 nm
0.05 0.25 0.50 0.75 1.00
B
Raw
Binned
2NA7
Fas(C178S)
Fas wt
Fas C178R
2NA7 Contour
Overlay
Fas C178S Clusters
Y (nm)
X (nm)

**Supplementary Figure 2 |** Fas assembly. **(A)** Comparison between the interaction matrix of Fas C178S NMR (PDB: 2na7) and the coarse grained molecular dynamic (CG-MD) simulations of Fas C178S, Fas wt and Fas C178R at 0.5 and 0.8 nm cut-off distance. The amino acid sequence corresponds to residues V254 to R274. Black arrowheads indicate conserved interactions whereas grey arrowheads indicate non-conserved interactions. Analysis was performed over the 10 NMR models (2na7) and between 3 to 6 µs in the CG-MD simulations. Scale corresponds to normalized number of contacts. **(B)** Comparison of the radial distribution of coarse grained Fas C178S NMR (PDB: 2na7) and the coarse grained molecular dynamic simulations of Fas C178S, Fas wt and Fas C178R. Top panels showed raw data. Middle panels show binned data, to allow better visualization of the main distribution spots. The trimeric assembly (T) of Fas C178S NMR structure is represented as an orange dotted triangle and overlapped with all the models. Lower panels show the same binned data in which it was overlapped the trimeric assembly of Fas C178S CD-MD model as green centred circles whose size correspond to the amount of data points in each cluster. Scales correspond to the 2D-density function from the scatter plot *XY*-coordinates.

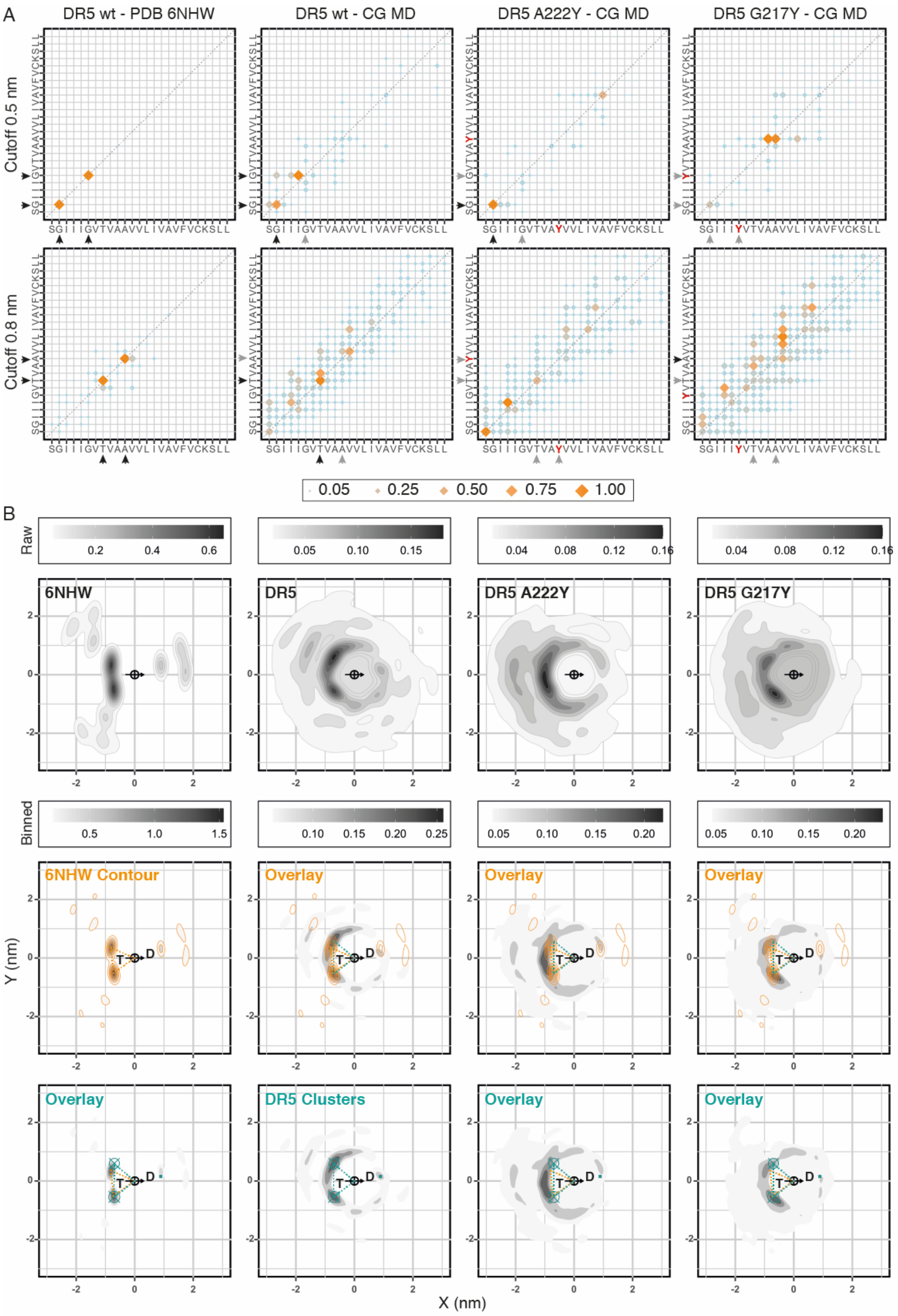
A
DR5 wt - PDB 6NHW
DR5 wt - CG MD
DR5 A222Y - CG MD
DR5 G217Y - CG MD
Cutoff 0.5 nm
Cutoff 0.8 nm
0.05
0.25
0.50
0.75
1.00
B
Raw
Binned
6NHW
DR5
DR5 A222Y
DR5 G217Y
6NHW Contour
Overlay
DR5 Clusters
Y (nm)
X (nm)

**Supplementary Figure 3 |** DR5 assembly. **(A)** Comparison between the interaction matrix of DR5 NMR (PDB: 6nhw) and the CG-MD simulations of DR5 wt, DR5 A222Y and DR5 G217Y at 0.5 and 0.8 nm cut-off distance. The amino acid sequence corresponds to residues S212 to L236. Black arrowheads indicate conserved interactions whereas grey arrowheads indicate non-conserved interactions. Analysis was performed over the 10 NMR models (6nhw) and between 3 to 8 µs of the CG-MD simulations. Scale corresponds to normalized number of contacts. **(B)** Comparison of the radial distribution of coarse grained DR5 NMR (PDB: 6nuw) and the coarse grained molecular dynamic simulations of DR5 wt, DR5 A222Y and DR5 G217Y. Top panels showed raw data. Middle panels show binned data, to allow better visualization of the main distribution spots. The dimeric assembly of DR5 NMR structure is indicated as (D) and the trimeric assembly (T) is represented as an orange dotted triangle overlapped with all the models. Lower panels show the same binned data in which it was overlapped the dimeric and trimeric assembly of DR5 wt CD-MD model as green centred circles whose size correspond to the amount of data points in each cluster. Scales correspond to the 2D-density function from the scatter plot *XY*-coordinates.

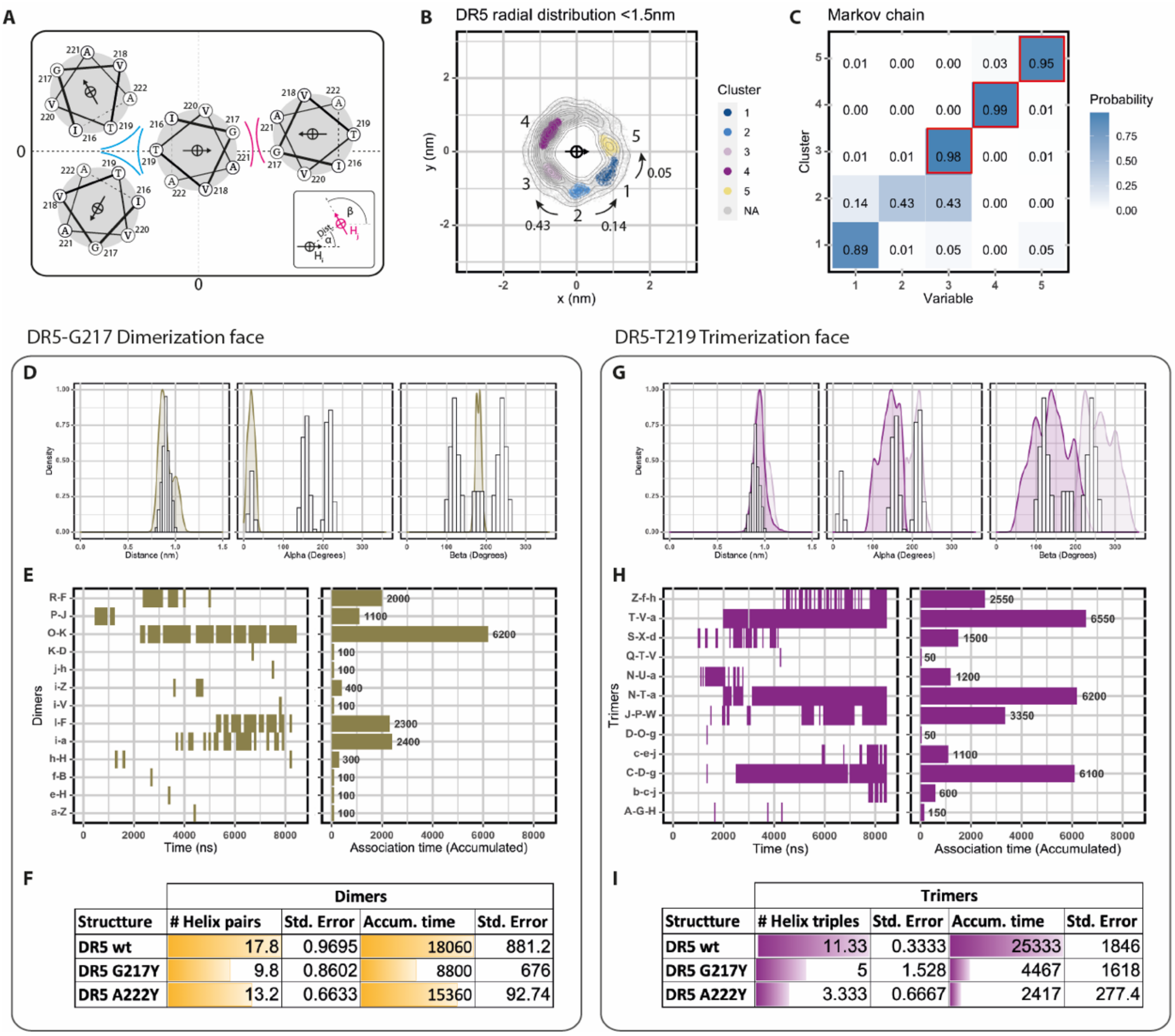


**Supplementary Figure 4 |** DR5 assembly, clustering and data analysis. **(A)** Schematic representation of the trimeric and dimeric associations of DR5. Trimeric and dimeric faces are shown in cyan and magenta colour lines respectively. In the center of each helix is depicted the centroid and the orientation vector. The location of the centroid of a pairing helix with respect to a central helix defines the alpha angle (inset). Beta corresponds to the angle between the orientation vectors of the pair of helices. **(B)** Radial distribution analysis of helices in the first interaction layer (distance <1.5nm). Dots correspond to each helix centroid coordinate in the XY plane that were clustered using DBSCAN and coloured by their cluster identity. Only the main clusters are shown. Arrows indicate the transition probability obtained by the markovian model (panel C). **(C)** Markov transition matrix. Intersections show the probability of transition from the "i" row (cluster) to the "j" column (variable) clusters. The diagonal shows the remaining probabilities. There is no absorbing state and the dimer (cluster 5) and trimers (clusters 3 and 4) have the highest remaining probabilities (red boxes). **(D-F)** The following analysis was applied using residue G217 as centroid. According to NMR data, this residue points toward the dimeric interface. Panel D shows frequency distributions of

distances, alpha and beta angles of the helices associated to cluster 5. The overlaid bars correspond to the NMR structure. Panel E shows a tile chart of unique dimers observed along the simulation (left) and the accumulated time of the corresponding dimers (right). Dimers were selected based on their cluster location. Panel F shows a table summarizing the average number of unique dimeric interactions and the average accumulated time. The averages were obtained by analysing NMR-like expected alpha and beta distributions using residues 217 to 221 as centroids. **(G-I)** Similar to panels D-F using residue T219 as centroid. According to NMR data, this residue points toward the trimeric interface.
